# Simultaneous Biochemical and Physiological Responses of the Roots and Leaves of *Pancratium maritimum* (Amaryllidaceae) to Mild Salt Stress

**DOI:** 10.3390/plants10020345

**Published:** 2021-02-11

**Authors:** Simona Carfagna, Giovanna Salbitani, Michele Innangi, Bruno Menale, Olga De Castro, Catello Di Martino, Thomas W. Crawford

**Affiliations:** 1 Dipartimento di Biologia, Università degli Studi di Napoli Federico II, 80126 Napoli, Italy; simona.carfagna@unina.it (S.C.); giovanna.salbitani@unina.it (G.S.); bruno.menale@unina.it (B.M.); olga.decastro@unina.it (O.D.C.); 2 Dipartimento di Scienze e Tecnologie Ambientali, Biologiche e Farmaceutiche, Università degli Studi della Campania Luigi Vanvitelli, 81100 Caserta, Italy; michele.innangi@unina.it; 3 Dipartimento di Agricoltura, Ambiente ed Alimenti, Università degli Studi del Molise, 86100 Campobasso, Italy; 4 Global Agronomy, LLC, Marana, AZ 85658, USA; globalagronomy@gmail.com

**Keywords:** amino acids, glutathione, OAS-TL, osmolytes, mineral nutrients, nucleotides

## Abstract

*Pancratium maritimum* (Amaryllidaceae) is a bulbous geophyte growing on coastal sands. In this study, we investigated changes in concentrations of metabolites in the root and leaf tissue of *P. maritimum* in response to mild salt stress. Changes in concentrations of osmolytes, glutathione, sodium, mineral nutrients, enzymes, and other compounds in the leaves and roots were measured at 0, 3, and 10 days during a 10-day exposure to two levels of mild salt stress, 50 mM NaCl or 100 mM NaCl in sandy soil from where the plants were collected in dunes near Cuma, Italy. Sodium accumulated in the roots, and relatively little was translocated to the leaves. At both concentrations of NaCl, higher values of the concentrations of oxidized glutathione disulfide (GSSG), compared to reduced glutathione (GSH), in roots and leaves were associated with salt tolerance. The concentration of proline increased more in the leaves than in the roots, and glycine betaine increased in both roots and leaves. Differences in the accumulation of organic osmolytes and electron donors synthesized in both leaves and roots demonstrate that osmoregulatory and electrical responses occur in these organs of *P. maritimum* under mild salt stress.

## 1. Introduction

Due to their sessile nature, plants undergo biotic and abiotic stress. By evolution of their metabolism, plants of *Pancratium maritimum* L. (Amaryllidaceae) have developed metabolic responses to mitigate environmental stresses and anatomical responses such as the bulb to protect sensitive parts of the plant, the leaves, and flowers, from extreme salt stress. Among abiotic stresses, salinity is a major constraint that restricts the growth and development of plants through, i.e., osmotic stress, cytotoxicity, nutritional imbalance, and oxidative stress [1]. Besides stressing plants in natural, saline environments, salinity can severely limit yield and productivity of crops, especially in the most productive areas of the world, such as the Mediterranean basin [2,3].

Halophytes are salt-resistant or salt-tolerant plants that complete their life cycle in saline habitats [4]. The tolerance of salinity is a complex phenomenon that involves various biochemical mechanisms and physiological adaptations, and *P. maritimum* represents an ideal model to understand metabolism contributing to resistance or tolerance to salt stress. While there are halophytes that can grow under salinity as extreme as 500 mM NaCl (~seawater salinity), optimal growth of many terrestrial, dicotyledonous geophytes that are exposed to mild salinity occurs within the range of concentrations of 50–250 mM NaCl [5,6,7].

*P. maritimum* is a bulbous, perennial geophyte typical of the Mediterranean region from the Black Sea to part of the Atlantic coast [8,9]. It grows wild on coastal sands and on coastal dunes, where mild salinity (~50–100 mM NaCl) is a key aspect of its optimal habitat. Coastal dune vegetation is exposed to severe atmospheric and edaphic stresses, which have caused these plants to evolve several mechanisms to counteract the stresses in order to grow and reproduce [10].

Although there is evidence that all plants, both sensitive and tolerant, use similar mechanisms in response to high salinity, little is presently known about the simultaneous mechanisms of salt tolerance of plants in their natural habitats. Salt tolerance of halophytes has been shown to be dependent on their capacity to compartmentalize toxic ions in the vacuole and to accumulate osmoprotectants, that are compatible solutes, in the cytoplasm [1,11]. Khan et al. [12] reported that the macronutrient sulfur is essential not only for plant growth but also for tolerance to salt stress. Many studies have investigated various mechanisms that protect plant cells from toxicity in the presence of high salinity [13,14], but these studies have not investigated S-containing compounds involved in salt stress responses. In all plants, essential compounds such as glutathione (reduced form, GSH; oxidized form, GSSG), hormones (e.g., ethylene, polyamines), vitamins, cofactors (e.g., biotin, thiamine, CoenzymeA), and many other secondary products are derived from the S-amino acid cysteine (Cys) [15,16,17]. Cys is required for the biosynthesis of abscisic acid (ABA), the phytohormone regulating responses to many abiotic stresses [18], and synthesis of Cys increases in many plants in response to various abiotic stresses [19,20,21].

The present study investigates, for the first time, roles of glutathione and *O*-acetylserine(thio)lyase (OAS-TL, EC 4.2.99.8), both involved in sulfur metabolism, in response to mild salt stress in a bulbous plant typically subjected to wide fluctuations of salinity in its natural environment. OAS-TL catalyzes Cys synthesis from sulfide and O-acetylserine, so we determined the enzymatic activity of OAS-TL in leaves and roots of *P. maritimum* within a range of salinity typical of its edaphic environment [9].

Therefore, the variable conditions of time of exposure and concentration of salt were applied in our experiments to present *P. maritimum* with a range of mild salt stress from a minimum of exposure to 50 mM NaCl for 3 days (d) to a maximum of exposure to 100 mM NaCl for 10 days. Changes in the concentration of some metabolites, such as osmolytes and glutathione, measured in different regimes of mild salt-stress and in the roots and leaves of the plants are described and discussed.

## 2. Results

### 2.1. Concentrations of Pyridine Nucleotides and Glutathione 

**Figure 1:**
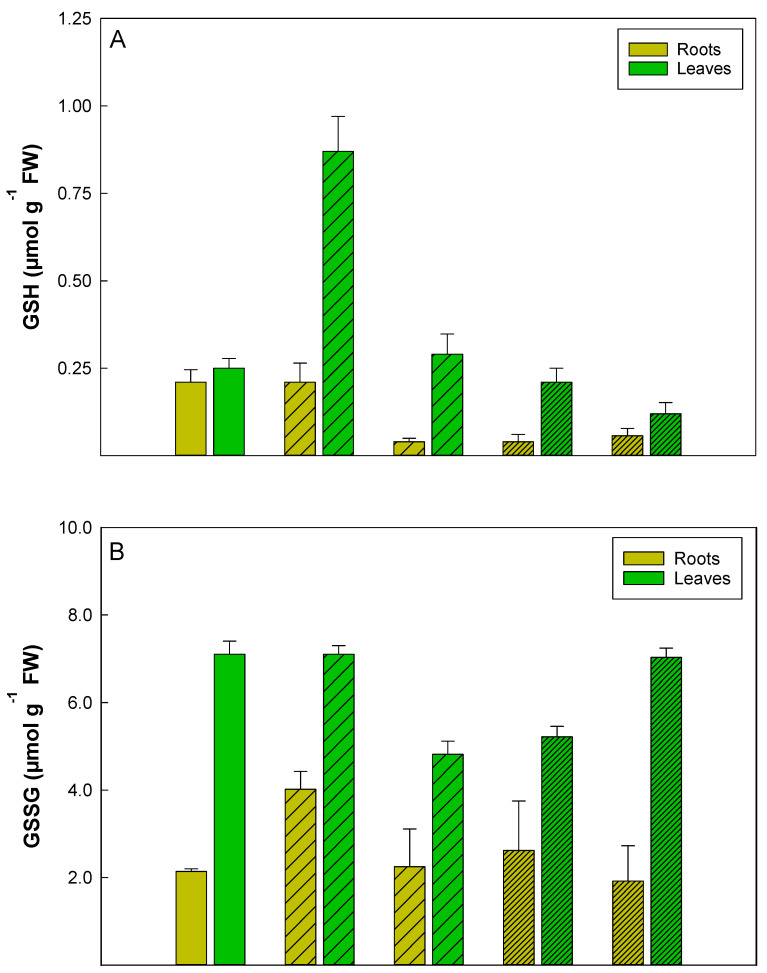
Glutathione (GSH) (**A**) and glutathione disulfide (GSSG) (**B**) concentrations (µmol g^−1^ FW) in roots (light green) and leaves (brilliant green) tissue of *P. maritimum* plants irrigated with 50 mM (wide striped) and 100 mM (narrow striped) of NaCl salt solution for 3 and 10 days. The values are means ± SD of three replicates.

Salt treatment with 50 mM and 100 mM NaCl provoked a slight increase in NADP^+^ and NADPH contents in roots and greater concentrations in the leaves. In both organs, there was a progressive decrease of the NADP^+^/NADPH redox ratio from about 2 to 1.25 in *P. maritimum* plants treated for 10 d with 100 mM NaCl (Table. 1). GSH content increased as much as ~3.2-fold in the leaves of plants treated with 50 mM NaCl after 3 days but there was no evidence of change in GSH concentration in the roots; at 10 d the GSH levels in the roots decreased by 80% compared to the control. The reduction in GSH concentration in the roots was more evident with the 100 mM NaCl treatment at both 3 and 10 d with a decrease of 80 and 70% respect to the control, respectively (Figure 1A). In the leaves, however, the concentrations of GSH after 50 mM treatment were always higher than the control. For example, at 3 ds after treatment, the GSH concentration reached 300% more than the control, and then decreased to 20% more at 10 days. The 100 mM treatment, however, resulted in a significant, albeit small, decrease in concentration of GSH in the leaves after 10 days with a reduction of 55% compared to the control.

**Figure 2:**
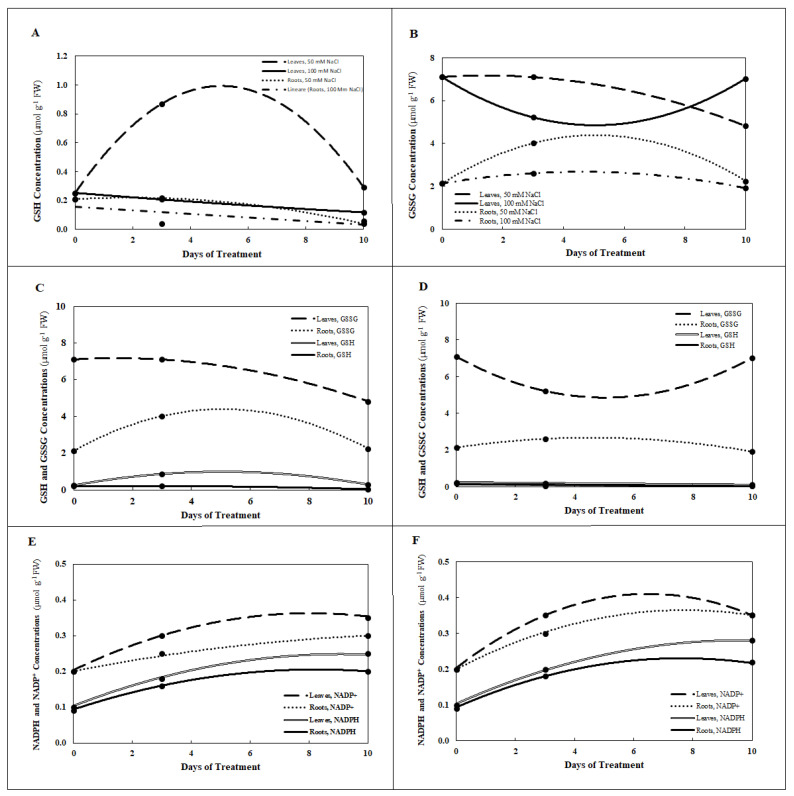
Estimates of concentration of reduced glutathione (GSH, **A**), oxidized glutathione (GSSG, **B**), GSH and GSSG in the roots and leaves treated with 50 mM NaCl (**C**), GSH and GSSG in the roots and leaves treated with 100 mM NaCl (**D**), and reduced nicotinamide adenine dinucleotide phosphate (NADPH, E), and oxidized nicotinamide adenine dinucleotide phosphate (NADP^+^, **F**) in roots and leaves of *P. maritimum* during 10 days of treatment with 50 mM NaCl (**E**) or 100 mM NaCl (**F**). Black dots represent the mean of three replicates.

During the 10-day treatment with 100 mM NaCl, the concentration of the GSSG in the leaves decreased and then returned to the level of control plants (Figure 1B). On the contrary, roots treated with 50 mM NaCl for 3 d accumulated more GSSG while the concentration of GSH was unchanged, with respect to the untreated plants. The concentrations of GSH and GSSG were greater in the leaves than in the roots with each NaCl treatment (Figure 2A,B). The concentrations of the oxidized compounds (GSSG and NADP^+^) were consistently greater than those of the corresponding reduced compounds (GSH and NADPH) for roots and leaves during 10 d for both 50 mM NaCl and 100 mM NaCl, as estimated by regression analysis (Figure 2C–F). Although NAD^+^/NADH ratio continued to be greater than 1 for the entire duration of the experiment, after 10 d of treatment with NaCl it decreased to 50%, both in the roots and leaves, indicating a proportionately greater increase in reduced NADH than oxidized NAD^+^ (Table 1).

### 2.2. OAS-TL Activity and Concentration of Protein

**Figure 3:**
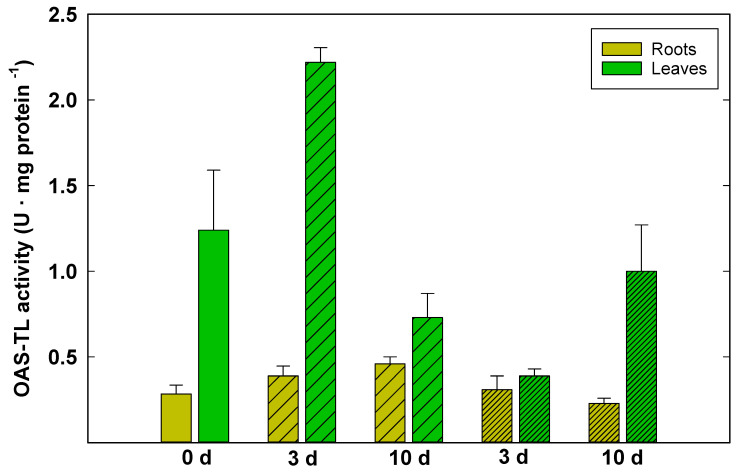
O-acetyl-l-serine(thiol)lyase (OAS-TL) activity (U mg protein^−1^) in roots (light green) and leaves (brilliant green) of *P. maritimum* plants irrigated with 50 mM (wide striped) and 100 mM (narrow striped) of NaCl salt solution for 3 and 10 days. The values are means ± SD of three replicates.

After 3 d of 50 mM NaCl treatment, the activity of OAS-TLs was enhanced strongly in the leaves and slightly in the roots. The 100 mM NaCl treatment resulted in a decrease in OAS-TL activity in the leaves at 3 d, followed by an increase at 10 d. In the roots, the 100 mM NaCl treatment slightly increased the activity of OAS-TLs at 3 d, and then the activity decreased at 10 d (Figure 3).

**Figure 4:**
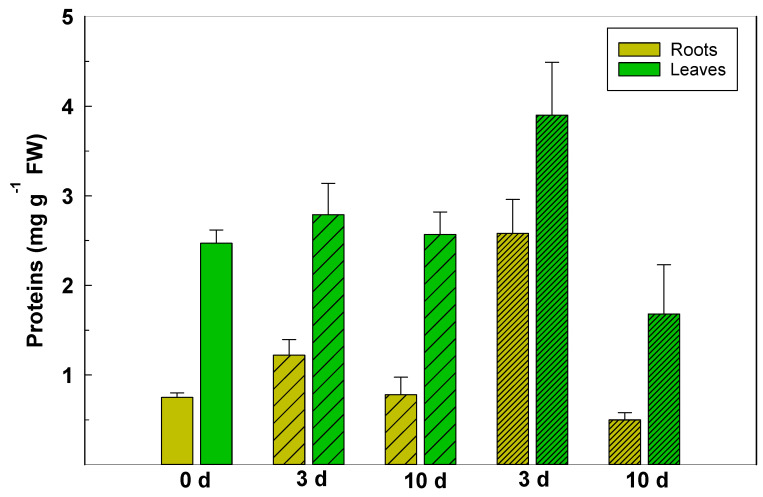
Total protein content (mg g^−1^ FW) in roots (light green) and leaves (brilliant green) tissue of *P. maritimum* plants irrigated with 50 mM (wide striped) and 100 mM (narrow striped) of NaCl salt solution for 3 and 10 days. The values are means ± SD of three replicates.

In *P. maritimum*, the effect of salt-stress on the concentration of protein in leaves and roots depended on both the NaCl concentration and the time of exposure to NaCl (Figure 4). After 3 d of treatment at 100 mM, the concentration of protein had significantly increased in roots and leaves, 3.3- and 1.6 times, respectively, with respect to the plants at 0 d before the start of treatments. After 10 d, the protein concentration decreased in both roots and leaves to about 70% of the respective concentrations of the non-treated.

### 2.3. Concentrations of Minerals in Leaves and Roots 

**Figure 5:**
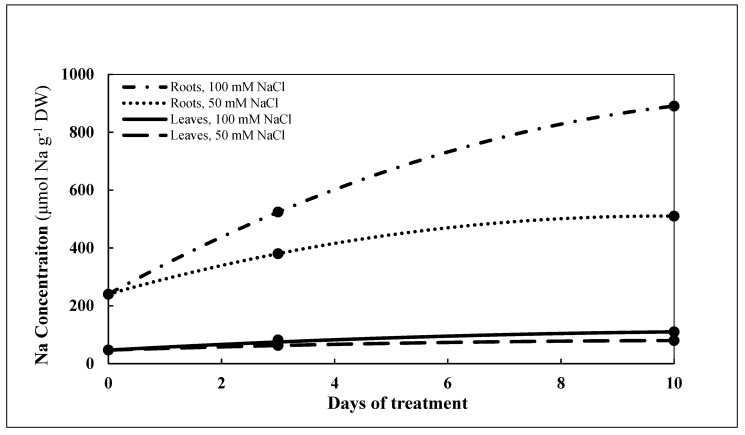
Estimates of concentration of Na (µmol g^−1^ DW) in roots and leaves of *P. maritimum* during 10 days of treatment with 50 mM NaCl or 100 mM NaCl. Black dots represent the mean of three replicates.

The concentrations of Na, K, Mg, Ca and Fe, Mn, Zn, and Cu of the roots and leaves of *P. maritimum* are shown in Table 2. The Na concentration of roots and leaves differed significantly between the two salt treatments during the 10-day experimental period. At 3 and 10 d after salinity treatment was begun, the Na concentration continued to increase and was much higher in the roots than in leaves (Figure 5).

The ratio of Na concentration of the leaves:roots (L:R) for the 50 mM NaCl treatment decreased from 0.19 (0 d) to 0.18 (3 d) to 0.16 (10 d), and the L:R ratio of Na content for the 100 mM NaCl treatment decreased from 0.20 (0 d) to 0.14 (3 d) to 0.12 (10 d) (Table S1). 

In both untreated and treated plants, the concentrations of Ca, K, and Mg were higher in leaves than in the roots. L:R ratio of dry-weight concentration of K, was 1.12 when the salinity treatments were begun at 0 d, 1.24 at 3 d, and 1.35 at 10 d with 50 mM NaCl treatment. The more stressful salinity treatment, 100 mM NaCl, resulted in the L:R K ratio increasing from 1.12 at 0 d to 1.43 at 3 d and to 2.37 at 10 d (Table S1). The two salinity treatments caused decreases in concentrations of K, Mg, and Ca both in leaves and roots. In the leaves, the decrease in the dry-weight concentration of these macronutrients does not exceed 30% in the most drastic salinity conditions of 100 mM at 10 days, i.e., 11, 27, and 30% for K, Mg, and Ca, respectively. In the roots, however, there is a more marked decline: 35, 40, and 62% for Mg, Ca, and K, respectively, while the dry-weight concentration does not exceed 25% at 50 mM 10 d (11%,18% and 25% for Ca, Mg, and K, respectively).

There was a significant increase in the concentration of Zn and Mn in leaves and roots in response to stress due to the increased NaCl concentration in the soil and increased exposure time. Increases in dry-weight concentrations of Mn and Zn of the roots and leaves were gradual with increasing salt concentration, reaching 40-50% more than the control at 10 days when treated with 100 mM NaCl. However, at 10 days, when treated with 100 mM NaCl, concentrations of Fe and Cu had significantly decreased in leaves (20 and 30%, respectively) and roots (33 and 31%, respectively). Iron is notable in the present study, being the one element whose L:R ratio of dry-weight concentration was least for the control (3.6) at 0 d, greater for the 50 mM NaCl treatment at 3 d and 10 d (3.9 and 4.1, respectively) and greatest for the 100 mM NaCl treatment at 3 d and 10 d (3.8 and 4.3, respectively) (Table S1). 

### 2.4. Concentrations of Free Amino Acids, Proline, and Glycine Betaine

**Figure 6:**
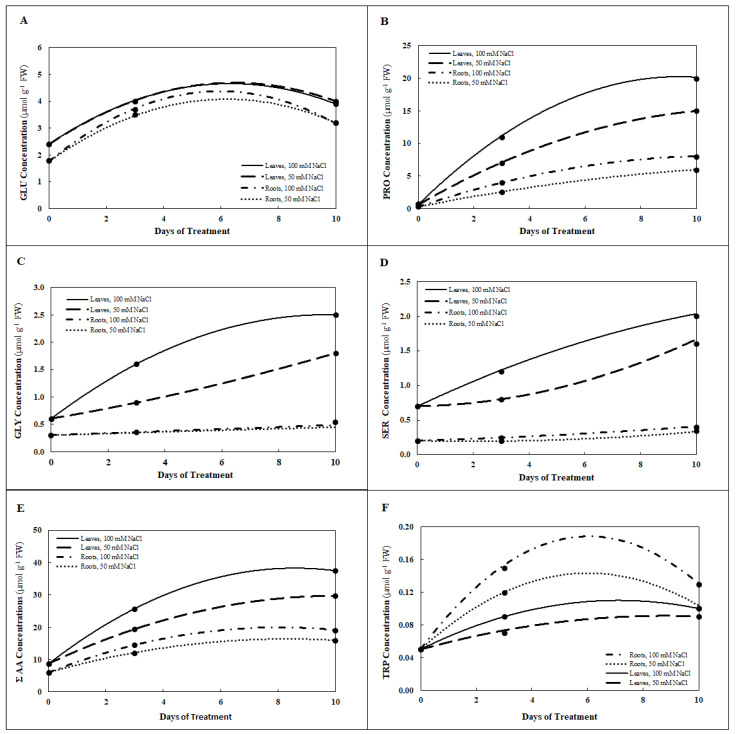
Estimates of concentrations (µmol g^−1^ FW) of glutamic acid (GLU, **A**), proline (PRO, **B**), glycine (GLY, **C**), serine (SER, **D**), the sum of amino acids in Table 3 (ΣAA, **E**), and tryptophan (TRP, **F**) in roots and leaves of *P. maritimum* during 10 days of treatment with 50 mM NaCl or 100 mM NaCl. Black dots represent the mean of three replicates.

An appreciable increase in the content of free amino acids (ΣAA, µmol g^−1^ FW) was detected in roots and leaves of *P. maritimum* in response to salt stress of both 50 and 100 mM NaCl (Table 3). Salt stress of 50 mM NaCl or 100 mM NaCl is associated with increases in the sum of concentrations of the 16 amino acids in the leaves from about 8.7 μmol g^−1^ FW in untreated plants to about 19.4 μmol g^−1^ DW with 50 mM NaCl treatment at 3 d and 37.5 μmol g^−1^ DW with 100 mM NaCl treatments at 10 d. A significant accumulation of proline was measured (greater than 25-fold with respect to the control) in both roots and leaves with salt treatment of 100 mM at 10 d (Table 3, Figure 6B). The concentration of proline in the leaves increased from 0.7 μmol g^−1^ FW at 0 d to 15 μmol g^−1^ FW with 50 mM and 20 μmol g^−1^ FW with 100 mM NaCl treatment after 10 d. Concentrations of both glycine and serine were greater in the leaves, compared to the roots, where there was little change in concentration, when *P. maritimum* plants were exposed to 50 mM NaCl or 100 mM NaCl for 10 d (Table 3, Figure 6C,D). Concentrations of glutamic acid were higher in the leaves than in the roots during 10 d of exposure to 50 mM NaCl or 100 mM NaCl, whereas the opposite occurred in the case of tryptophan (Table 3, Figure 6A,F).

**Figure 7:**
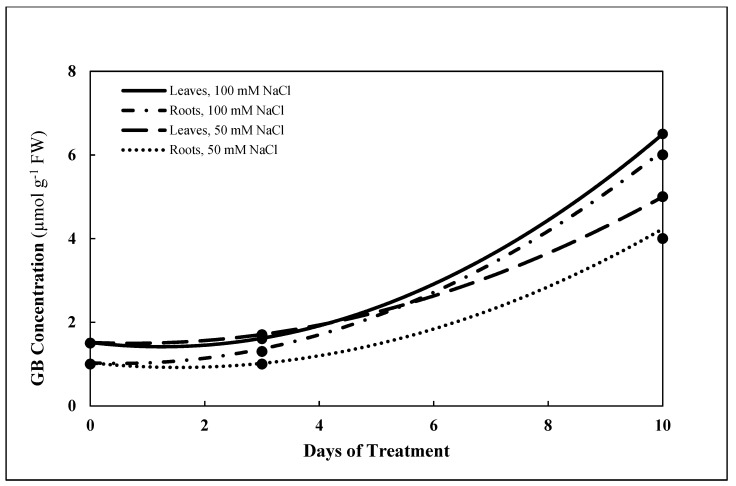
Estimates of concentration (µmol g^−1^ FW) of glycine betaine (GB) in roots and leaves of *P. maritimum* during 10 days of treatment with 50 mM NaCl or 100 mM NaCl. Black dots represent the mean of three replicates.

The amino acid derivative glycine betaine (trimethylglycine) increased in roots as well as in leaves, reaching maximum concentrations in both organs after salt treatment for 10 d (Table 3). The concentration of glycine betaine in the leaves increased from 1.5 μmol g^−1^ FW at 0 d to 5.0 μmol g^−1^ FW and to 6.5 μmol g^−1^ FW at 10 ds with 50 and 100 mM NaCl treatments, respectively (Figure 7). Concentrations of the amino acids determined and of glycine betaine also increased in the roots in response to salinity.

### 2.5. Principal Component Analysis

**Figure 8:**
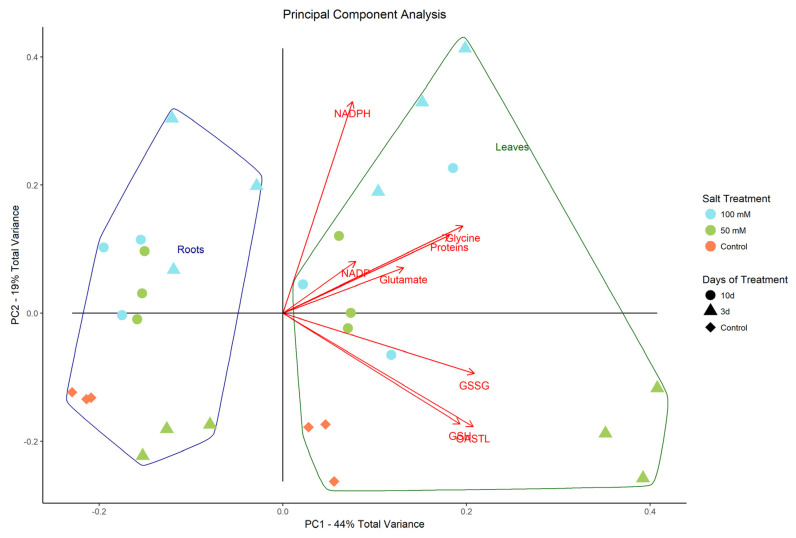
Principal Component Analysis, or PCA (PC1 and PC2: 63%): PCA biplot displaying distribution of treatments grouped according to different salt concentration (50 and 100 mM) and days of exposure (3 and 10 days).

The first two principal components (PCs) explain 63% of total variance (Figure 8). The plot clearly shows a separation associated mostly with type of plant tissue (leaves on the right and roots on the left of PC1), and a secondary split is seen on PC2, associated with salinity and days of treatment. Despite the overall higher activities in leaves compared to roots (explained by the split on PC1), the differences on PC2 were comparable between leaf and root tissues. In detail, the control at 0 d and 50 mM NaCl treatment after 3 d showed higher GSSG, GSH, and OAS-TL activities. The 100 mM NaCl treatment at 3 and 10 d and the 50 mM NaCl treatment after 3 d all exhibited higher concentration of Gly, proteins, Glu, and NADP^+^. Noticeably, the NADPH concentration peaked at 3 d in the 100 mM treatment both in roots and leaves.

## 3. Discussion

In general, plants respond to salinity with an increase in abscisic acid, a biomolecular mediator in plant responses to salt stress, and with a decrease in stomatal aperture to maintain internal water content. As a result, CO_2_ fixation by the Calvin cycle is limited, with a consequent reduction of NADP^+^ stromal concentration. Under the mild stress applied, no obvious physiological alterations such as growth inhibition or dehydration symptoms were observed, but our results show a metabolic response, namely a decrease of the NADP^+^/NADPH redox ratio in leaves of salt stressed *P. maritimum* plants. These results are consistent with the ability of the photosynthetic cell, under conditions of mild stress, to respond to a significant decrease in the NADP^+^/NADPH ratio, indicating an altered synergy in the redox balance between the light and dark phases of photosynthesis, triggering the pseudo-cyclic electron transport chain. In fact, a decrease in the NADP^+^/NADPH redox ratio in leaves of salt-stressed plants, most likely due to a limitation of CO_2_ fixation, could increase the formation of reactive oxygen species (ROS) and the activity of biochemical processes involved in oxidative stress detoxification. In plants, adverse environmental conditions such as drought, high and low temperatures, or salinity promote the overproduction of ROS resulting in disruption of the intracellular redox equilibrium [22]. To counteract the harmful effects of ROS, plants maintain cellular redox homeostasis by utilizing many compounds that are antioxidative electron donors. Antioxidants are molecules with a low molecular mass capable of inhibiting or quenching free radical reactions, thereby preventing damage to cellular molecules.

Among antioxidants, the tripeptide glutathione plays a key role in responding to environmental stress such as salinity and is a major factor responsible for salt tolerance of the halophyte [23]. In addition, under environmental stress, the reduced and oxidized forms of glutathione (GSH and GSSG) may also have role in regulating stress-related marker genes at the transcriptional level [23]. The total glutathione concentration increased in *P. maritimum* plants treated with salt. The reduced form of glutathione, GSH, may have protected the plants from salt stress, since the concentration of GSH increased more than three-fold in the leaves of plants under moderate stress of 50 mM NaCl for 3 d. At the same time that GSH increased in the leaves of these plants, the concentration of GSSG increased in the roots. As the oxidized form GSSG tended to increase in the roots under moderate salt stress, the reduced form GSH in the roots tended to decrease as time progressed during the experiment.

In the treated plants, the increase in total glutathione was more evident in the roots with a greater proportion in the oxidized fraction, confirming a more energetic response to stress in these organs, with respect to the leaves. Nicotinamide adenine dinucleotide phosphate is the oxidative-reductive cofactor of the enzyme glutathione reductase. This enzyme, in fact, regenerates GSH from GSSG through the electrons transferred from the reducing agent, NADPH. This would explain the increase of GSH in the leaves that could also be interpreted as a drain of reduction equivalents to avoid the trigger of a pseudo-cyclic electronic transport, such as commonly occurs in osmotic stress conditions. The maintenance of a high GSH to GSSG ratio in the leaves (Figure S1) through a NADPH-dependent plastid GSSG reductase [24,25,26] could be a factor that improves the salt tolerance in *P. maritimum* plants by diminishing oxidative stress in these organs. In fact, the loss of productivity in all plants under salt stress is generally associated to the overproduction of ROS and the change of redox state of the cell [12,23,24,25]. Regardless of level of sodium chloride (50 or 100 mM), the concentration of GSSG was higher than that of GSH in the leaves and in the roots, suggesting that (1) reduced glutathione (GSH) served as a prominent, protective electron donor in response to oxidative stress in both organs and (2) in *P. maritimum*, glutathione appears to be a key factor contributing to salt-tolerance. Moreover, the concentration of GSSG was higher with exposure to 100 mM NaCl than with exposure to 50 mM NaCl in both leaves and roots, indicating a more robust presence of glutathione in the leaves where photosynthesis requires protection from ROS to function.

In plants subjected to mild salt stress (50 mM NaCl for 3 d), the increase of glutathione content in the leaves was supported by enhancement of OAS-TL enzymes, as indicated by Spearman positive correlation (Figure S2). In plants, OAS-TL carries out the cysteine biosynthesis from O-acetyl-L-serine [27,28]. Among proteins up-regulated by salt-stress, the OAS-TLs enzyme seems to have an important role. In leaves, after 3 d of 50 mM NaCl treatment, the high activity of OAS-TL corresponds to a relatively high concentration of GSH. In roots, the salt treatment slightly increased the activity of OAS-TL at the concentration of 50 mM NaCl and did not affect the activity of OAS-TL at the concentration of 100 mM NaCl. Moreover, OAS-TL gene expression in *P. maritimum* in response to mild salt stress [29] has been reported as being highest in leaves, followed by lesser responses in the roots and bulbs. Unlike other plants where higher amounts of OAS-TL transcripts occur in the roots under stress [19], OAS-TL mRNA expression in roots of moderately salt-stressed *P. maritimum* remained almost unchanged [29].

The OAS-TL mRNA expression and activity enhancement in leaves might be explained by increased demand for cysteine needed for glutathione synthesis. The high demand for cysteine during salt stress is also in agreement with the role of sulfur-containing compounds that function as osmolytes or antioxidants [30]. This means that the synthesis of cysteine and of thiol compounds such as glutathione in *P. maritimum* plants represents an essential early reaction mechanism following the exposure of the plants to salinity, decreasing gradually with time.

Metabolism of total protein in *P. maritimum* was profoundly affected by salt. The significant change in protein contents in both the roots and the leaves points to an imbalance between anabolic and catabolic processes. Such an imbalance in response to saline stress could occur either from up-regulation or down-regulation of the synthesis of proteins [31]. The higher concentrations of protein in the salt-stressed plants, compared to the untreated plants, may possibly be an initial defense against osmotic stress, and after 10 d, the decreased concentrations of protein in the fresh tissue may be a result of disintegration of stress-damaged proteins. Furthermore, the concentrations of NAD^+^ and NADH in the roots and leaves provide a measure of the oxidative metabolism of carbon bonds which are the main source of reduction equivalents. The energy required by the cell to ensure a homeostatic reaction mainly through membrane ATPase activity and synthesis of osmolytes and free amino acids in the roots can only be provided by the oxidation of glucose. This cellular energy flow through glycolysis and the Krebs cycle increases ATP levels and the NADH/NAD^+^ ratio.

According to Morant-Avice et al. [32], proteins are very susceptible to severe salt-toxicity because their synthesis is dependent on physiological potassium (K^+^) and because absorption of K^+^ is inhibited by excess of sodium (Na^+^) and chloride (Cl^-^) [33]. Saline stress also causes a physiological imbalance between Na^+^ and K^+^, an essential macronutrient that is crucial for protein synthesis [33,34]. 

Our data clearly show that *P. maritimum* is able to protect its photosynthetic organs from toxic levels of Na by, at least partially, accumulating relatively large concentrations of Na in the roots. This is a general feature of salt-tolerant monocotyledonous, which largely base their mechanisms of salt tolerance on blocking the transport of toxic ions to the aboveground plant organs [5]. During the 10-day experimental period with treatments of 50 mM or 100 mM NaCl, Na accumulated in the roots, i.e., 270 μmol Na g^−1^ DW and 650 μmol Na g^−1^ DW, respectively, more than the concentration of Na before the treatments were begun. Accumulation of Na in the leaves gradually appeared, being limited to 63 μmol Na g^−1^ DW more than untreated plants after 10 d of treatment with 100 mM NaCl. These results indicate that *P. maritimum* accumulated and stored large quantities of Na in the roots, probably in the vacuoles of parenchymal cells [35] thereby inhibiting Na transfer to the leaves. In plants sampled before treatment was begun and during treatment at 3 and 10 d, Ca, K, and Mg contents were higher in leaves than in the roots, indicating that these three elements, after absorption by the roots, were being translocated to the leaves where they could contribute to photosynthetic metabolism and other metabolic processes. Unlike other plants where the decrease in Ca and Mg concentrations was significant and started at lower salinity level (25 mM NaCl) [36], in *P. maritimum*, Ca and Mg concentrations in both roots and leaves remain relatively unchanged during treatment with a 25% drop only after 10 d at 100 mM NaCl; this decrease of 25% was probably not sufficient to generate physiological alterations to the plant.

The cations K^+^, Na^+^, NH_4_^+^, and H^+^ directly and indirectly depress Ca^2+^ and Mg^2+^ uptake and distribution [37] probably acting as antagonists to Ca^2+^ and Mg^2+^ uptake. Moreover, K^+^ plays essential roles in enzyme activation and plasma membrane protein synthesis. Though, on the whole, Ca^2+^ and Mg^2+^ were the less affected cations, compared to the monovalent cations, a significant reduction in calcium could make the plasma membrane more permeable, resulting in less well-controlled influx and efflux. Salinity can increase the incidence of calcium-related physiological disorders either by competition between Na^+^ and Ca^2+^ during uptake, or by decreasing the soil water potential and thus root pressure [38]. Although numerous studies have addressed the effect of Mg deficiency on biomass and photosynthetic CO_2_ assimilation, in *P. maritimum* the Mg concentrations remain substantially unchanged during treatment and there are no effects of Mg deficiency. In *P. maritimum,* the L:R ratio of total dry-weight concentration of K increased in plants salt-stressed with 50 mM NaCl at 3 and 10 d, respectively. The more stressful salinity treatment, 100 mM NaCl for 10 d, resulted in a strong K L:R ratio increase. 

The increase in the accumulation of ions like Na^+^ and Cl^−^ caused toxicity in the roots of *P. maritimum* plants, since high concentrations of Na^+^ plus Cl^−^ in soil have been shown to decrease the uptake and assimilation of K^+^ and Ca^2+^, resulting in a reduction of photosynthesis, stomatal conductance, and chlorophyll content like in other plants [39,40]. The uptake and assimilation of micronutrients under salt stress depends upon genotype and salinity level [33]. The availability of micronutrients under salt stress is affected by many factors such as micronutrient solubility, and the pH and redox potential of the soil solution [38,41].

There was a significant increase in the concentration of Zn and Mn in leaves and roots in response to stress due to increased NaCl concentration and exposure time. The increases in dry-weight concentrations of Mn and Zn in the roots and leaves were gradual with increasing salt concentration. At 10 d, when plants were treated with 100 mM NaCl, concentrations of Fe and Cu had significantly decreased in leaves. Iron is notable in the present study, being the one element whose L:R ratio of dry-weight concentration increased with NaCl concentrations and duration of treatment. However, the foliar concentration of Fe at 10 days with 100 mM NaCl treatment, the most stressful combination of duration and concentration, indicates that Fe could still be sufficient to guarantee metabolic functions such as chlorophyll synthesis, photosynthesis, respiration, and normal functioning of many enzymes of which Fe is a cofactor [42]. Similarly, Mn also plays an important role as an activator of various enzymes such as decarboxylase, dehydrogenase, superoxidase, and phosphatase, as well as functioning as a constituent of photosystem II protein in photosynthesis. Finally, the micronutrients Fe, Mn, and Zn improve plant tolerance against salt stress by activating the synthesis of proline, an osmoprotectant that helps to tolerate the effect of salts [43].

Total amino acid content in response to salt treatment has been reported to increase in several plant species [44]. An appreciable increase in the concentrations of free amino acids was detected in roots and leaves of *P. maritimum* in response to mild salt stress at both 50 and 100 mM NaCl. Glutathione is a tripeptide formed by three amino acids, Cys, Gly, and Glu. In both roots and leaves of plants treated with 100 mM NaCl, the concentrations of Gly were greater, with respect to untreated plants. Also, the concentrations of Glu, Gln, and Ala were higher in roots and leaves of plants salt stressed, compared to those untreated, suggesting a general unbalancing of nitrogen assimilation [35].

Concentrations of tryptophan were higher in the roots than in the leaves during the entire 10 d of exposure to 50 mM NaCl or 100 mM NaCl. In plants, tryptophan is the primary precursor of the predominant endogenous auxin (indole-3-acetic acid). One of the hormones involved in plants’ response to salt stress response is auxin. Ribba et al. [45] reported that increased auxin levels can lead to a reduction in uptake of toxic ions by plants under salt stress. Therefore, our data may indicate that the relatively high concentrations of tryptophan in the roots of *P. maritimum* in the roots, compared to the leaves, are a part of complex tolerance mechanisms protecting *P. maritimum* from adverse effects of moderate salt stress.

These findings, in addition to the confirmation of the important role of the pool of free amino acids in osmoregulation, are consistent with the reports of large increases of protein content in the early stages of salt stress.

The increase in free amino acids was mainly due to the intracellular increase of proline in both roots and leaves that was most pronounced with salt treatment of 100 mM for 10 d. Proline plays important protective roles in the acclimation to salinity stress by osmotic adjustments and protecting plants’ subcellular structures [46]. Besides functioning as a compatible solute [44], proline, as it accumulates during salt stress, may be involved as signal-inducing for dehydrin synthesis in roots and stems or may be associated with stress-responsive genes [47]. In addition to proline, glycine betaine, another compatible solute having an important role in tolerance to osmotic stress, increased in roots as well as in leaves, but only after a prolonged salt treatment for 10 d. All plant cells, when kept in a low water potential environment, increase the internal concentration of osmolytes. To avoid shrinkage and/or swelling of intracellular organelles, equivalent concentrations of such compounds must be kept in the intracellular compartments [35]. The similar increase of osmolarity, as a result of storage of soluble organic nitrogen compounds (the amino acids proline and glutamic acid and the amino acid derivative glycine betaine) in the leaves and roots of salt-stressed *P. maritimum*, suggests that different osmo-regulatory responses may also depend on different osmotic stresses in different plant organs and organelles. These findings, in addition to the confirmation of the important simultaneous roles of the amino acids in osmoregulation, are consistent with the reported high increase of protein content in *P. maritimum* in the early stages of salt stress. On the other hand, after 10 d with relatively severe stress conditions (100 mM), the increase in free amino acids in both roots and leaves (56 and 66%, respectively), could be partially attributed to catabolic processes such as protein hydrolysis or proline conversion, and partially to anabolic processes such as their biosynthesis. 

The simultaneous increases of the concentrations of the intracellular free amino acids, especially of proline, and their osmotic properties could result in further, simultaneous metabolic interactions in the defense of osmotic stress. For example, the synthesis of plastid proline [48] occurs through a reduction process of Glu, and reduction of Glu involves an inevitable drainage of reduction equivalents from the plastid stroma, reducing the damage of an osmotic stress, such as the triggering of a pseudo-cyclic electron transport and ROS generation. Moreover, the simultaneous increase in the concentration of intracellular Gly and Ser (400 and 300% more than untreated plants) in the leaves of plants irrigated with 100 mM NaCl for 10 d expresses an ongoing photorespiratory activity. As is well known, the amino acids indicating a photorespiratory activity are Gly, which is a constituent of glutathione, and Ser, which is the precursor of *O*-Acetylserine, substrate of OAS-TL enzyme. *O*-acetylserine is biosynthesized by acetylation of Ser by the enzyme serine acetyltransferase [49]. The enzyme *O*-acetylserine (thiol)-lyase, using sulfide sources, converts this ester into cysteine, releasing acetate. From a careful reading of the experimental data, it is possible to rationally conclude that photorespiration can biochemically support the metabolism of sulfur and the synthesis of glutathione, whose reduction is catalyzed by NADPH-dependent chloroplastic glutathione reductase enzyme.

The present investigation provides glimpses of many elemental and molecular components that act together simultaneously in the complex metabolic and physiological system of the *P. maritimum.* The results of the present investigation demonstrate many aspects of metabolism that *P. maritimum* has evolved to defend itself against the metabolic instability that can result from increased concentration of NaCl in the root zone.

## 4. Materials and Methods

### 4.1. Plant Growth and Treatment

*P. maritimum* plants were collected from dunes in Cuma (near Naples, Italy) and were cultivated in pots with their original, sandy substrate. The plants were supplied every two days (d) with 200 mL of Hoagland solution [50]. After 7 d of pre-treatment with Hoagland solution, the plants were divided into 3 groups: (1) Control; (2) Hoagland solution plus 50 mM NaCl; and (3) Hoagland solution plus 100 mM NaCl. Three replicate control plants were harvested before treatments were applied. At 3 and 10 d after treatments were begun, three replicate plants were harvested for each of the two salinity treatments. Leaves and roots were harvested from the bulb of each plant. These two organs excised from plants were washed with deionized water, fresh weight was determined, and then samples of the organs were blot-dried and frozen in liquid nitrogen before being used for enzyme assays and biochemical measurements. Other samples of the roots and leaves were air-dried in a heater with constant heat control at 40 °C to constant dry weight for mineral analysis. 

### 4.2. Analyses of Amino Acids, Proline, Glycine Betaine, Pyridine Nucleotides 

Aliquots of fine, powdered samples of leaves (200 mg FW) and roots (350 FW) from five plants per treatment were used for chemical determination of amino acids and pyridine nucleotides. The samples were separately suspended in 2 mL of ethanol/water (80/20 *v*/*v*). After 30 min, the suspension was collected and centrifuged. The supernatant was used to determine the concentration of amino acids and nucleotides on a fresh-weight basis. The primary amino acids were determined by autosampler-assisted pre-column derivatization by o-phthaldialdehyde (OPA), separation by C 18 reverse-phase high-performance liquid chromatography (HPLC) and fluorescence detection (excitation at 340 nm and emission at 450 nm) [35]. Proline was determined by HPLC as fluorescent 9-fluorenylmethoxycarbonyl derivative (P-FMOC-carbamate) on sample extracts that were previously derivatized by OPA reagent to remove the primary amino acids and fluorometrically detected using excitation at 254 nm and emission at 315 nm [35]. The nucleotides were determined by reverse-phase high-performance liquid chromatography (HPLC). Column: SUPELCOSIL LC-18-T (3 µm particles) with guard column. Mobil phase: A = 0.1 M KH_2_PO_4_ plus 4 mM tetrabutylammonium hydrogen sulfate pH 6; B = A: methanol, 70:30, pH 7,2. Flow rate 1,5 mL/ min Det: 254 nm. Gradient program: Time (min) 0% B 0; 2.5-0; 5-30; 10-60; 13-0; 17-0; 18-100 [51]. The chemical analyses were repeated three times. In the analysis of each compound, the measurements were repeated using at least three different samples for each treatment. The results are given as the mean ± SD (n = 3) of replicates within each experiment.

### 4.3. Glutathione Determination

Fresh leaves and roots (1 g) from five plants of *P. maritimum* were crushed in mortars with liquid nitrogen, and 2 mL of 5% sulfosalicylic acid were added to the powdered tissue. The homogenates were centrifuged at 12000× *g* for 15 min at 4 °C. Reduced, disulfhydryl glutathione (GSH) and oxidized glutathione disulfide (GSSG) were determined according to Carfagna et al. [17]. The extract (100 µL) was added to 600 µL of reaction buffer (0.1-M Na-phosphate, pH 7.00, and 1-mM of ethylenediaminetetraacetic acid (EDTA), 40 µL of 0.4% 5,50 -dithiobis-(2-nitrobenzoic acid) (DTNB) (5,50 -dithiobis-2-nitrobenzoic acid), and 400 µL of distilled water. The GSH content was determined at 412 nm after 5 min. Then, 50 µL of 0.4% reduced nicotinamide adenine dinucleotide phosphate (NADPH) and 1 µL of glutathione reductase (GR) (0.5 U) were added to the reaction mixture, and the content of total glutathione (GSH plus GSSG) was determined at 412 nm after 30 min of incubation at room temperature. Amounts of GSH and GSSG were expressed in µmol g^−1^ FW.

### 4.4. O-acetyl-l-serine(thiol)lyase (OAS-TL) Enzymatic Activities and Protein Determination

One gram of fresh leaves and roots of *P. maritimum* was crushed by mortar in liquid nitrogen, and to the powder, 2 mL of lysis buffer (50 mM phosphate-buffer pH 7.5, 10 μM pyridoxal-5′-phosphate, and 1 mM dithiothreitol) were added. Lysates were cleared by centrifugation at 12000× *g* for 15 min at 4 °C. The supernatant represented the crude extract. The enzymatic activity of OAS-TL was measured according to Carfagna et al. [20]. The OAS-TL activity was related to the total soluble protein content of the samples. Protein amounts were determined using the reagent Protein Assay (Bio-Rad Laboratories GmbH, Vienna, Austria) based on the Bradford method [52] with bovine serum albumin as the standard.

### 4.5. Plant Mineralization and Mineral Element Analysis

Aliquots of 0.5 g of the air-dried roots or leaves were acid digested in a mixture of 6 mL of 60% HNO_3_ and 1 mL of 30% H_2_O_2_ at 80 °C for 40 min. The digestate was filtered and brought up to 50 mL with ultrapure water (Merk-Millipore Aquatron). The mineral elements were detected by Multi-element measurement in the (ICP-OES) instrument “Inductively Coupled Plasma” Varian Vista [35].

### 4.6. Statistical Analysis

Data were evaluated by analysis of variance (ANOVA) using SPSS Statistics ver. 20. Differences among means were determined using the Scheffè’s test. Data are presented as the mean ± SD and a value of *p* < 0.05 was used to indicate statistical significance. Nearest neighbor clustering with Euclidean distance and Pearson and Spearman correlation coefficients, using the two-tailed test of significance, was also performed to analyze relations among OAS-TL activity, total soluble protein, GSH and GSSG, amino acids, and several metabolites. The results are shown in the Supplementary Materials (Figure S1). Correlation among variables was tested by means of Spearman’s rank correlation coefficient. The overall pattern of variations between tissues (roots vs. leaves), salinity, and days of treatment was analyzed by means of a between-group Principal Component Analysis (PCA).

Curves in Figure 2, Figure 5, Figure 6 and Figure 7 represent estimates based upon regression analysis of the measured concentrations at 0, 3, and 10 d using the quadratic equation, a*x*^2^ + b*x* + c as a model using Microsoft Excel software, with one exception. In the case of GSH in the roots treated with 100 mM NaCl, the concentration was estimated with the model a*x* + b, because modeling with a*x*^2^ + b*x* + c produced some slightly negative values for the estimate of GSH concentration between 3 and 10 d.

### 4.7. Modelling of Dynamic Concentrations with Regression Analysis

Using regression analysis to model data taken at different points in time enables estimation of continuous, dynamic aspects of biochemical responses to mild stress by NaCl from 0 d through 10 d. The primary data on which statistical data presented in Table 1, Table 2 and Table 3 are based were used to provide continuous estimates of the concentrations of GSH, GSSG, NADPH, NADP+, Na, and the amino acids GLU, PRO, GLY, SER, TRP, and Σ amino acids. Second-order quadratic and first-order linear models were tested, and the quadratic model provided the best fit for all but one of the curves presented in Figure 2, Figure 5, Figure 6 and Figure 7. Estimates using the second-order model are based upon regression analysis of the measured, replicated concentrations at 0, 3, and 10 d using the quadratic equation, a*x*^2^ + b*x* + c, where *x* = time measured in days. In the one case where a first-order equation was chosen, GSH in the roots treated with 100 mM NaCl, the concentration was estimated with the model a*x* + b, because modeling with a*x*^2^ + b*x* + c produced some slightly negative values for the estimate of GSH concentration between 3 and 10 d.

## 5. Conclusions

Salt stress of *P. maritimum* applied with either 50 mM NaCl and 100 mM NaCl treatment, as measured at 3 and 10 d, compared to untreated plants at 0 d, resulted in accumulation of Na in the roots and relatively little translocation and accumulation of Na in the leaves. Potassium, Ca, Mg, and Fe were, on the other hand, translocated from the roots and accumulated at higher concentrations in leaves than in the roots after application of salt stress, as measured at 3 d and 10 d. Metabolism of *P. maritimum* in terms of total proteins and free amino acids was profoundly affected by salt stress depending on the concentration of NaCl and the duration of treatment. The simultaneous, large increases in concentrations of amino acids, particularly proline, and the amino acid derivative glycine betaine, indicate production of osmotic compounds in the leaves and roots in response to the large influxes of Na that was sequestered mainly in the roots. Our results show that OAS-TL activity was affected by salt, appearing after 3 d of treatment, and disappearing later (10 d), when other adaptation mechanisms such as higher concentrations of osmotic compounds are evident. Simultaneous synthesis of S-containing compounds, such as glutathione and its constituent amino acids, was supported by photorespiration, which could have a significant function in sulfur metabolism under conditions of osmotic stress due to increased salinity. High concentrations of GSSG and NADP^+^ that accumulated in response to treatment with NaCl indicate that biosynthesis of glutathione and nicotinamide adenine dinucleotide phosphate are normal, simultaneous responses of *P. maritimum* to oxidative imbalance resulting from salt stress.

## Figures and Tables

**Table 1:** Pyridine nucleotides (µmol g^−1^ FW) in roots and leaves tissue of *P. maritimum* plants irrigated with 50 and 100 mM of NaCl salt solution for 3 and 10 days.

	Control	50 mM	NaCl	100 mM	NaCl
Days	0	3	10	3	10
Roots (µmol g^−1^FW)					
NADP^+^	0.20 ± 0.03^Aa^	0.25 ± 0.04^Aa^	0.30 ± 0.04^Aa^	0.30 ± 0.03^Aa^	0.35 ± 0.04^Aa^
NADPH	0.09 ± 0.03^Aa^	0.16 ± 0.03^Bb^	0.20 ± 0.03^Bb^	0.18 ± 0.02^Bb^	0.22 ± 0.04^Bb^
NAD^+^	0.40 ± 0.03^Aa^	0.55 ± 0.04^Ab^	0.60 ± 0.04^Ab^	0.55 ± 0.04^Ab^	0.65 ± 0.04^Ab^
NADH	0.15 ± 0.02^Aa^	0.30 ± 0.03^Bb^	0.40 ± 0.03^Bb^	0.32 ± 0.03^Bb^	0.50 ± 0.04^Bc^
Leaves (µmol g^−1^FW)					
NADP^+^	0.20 ± 0.03^Aa^	0.30 ± 0.03^Ab^	0.35 ± 0.02^Ab^	0.35 ± 0.03^Ab^	0.35 ± 0.02^Ab^
NADPH	0.10 ± 0.03^Aa^	0.18 ± 0.03^Ab^	0.25 ± 0.003^Ac^	0.20 ± 0.02^Ab^	0.28 ± 0.03^Ac^
NAD^+^	0.40 ± 0.06^Aa^	0.60 ± 0.03^Ab^	0.55 ± 0.03^Ab^	0.50 ± 0.02^Ab^	0.50 ± 0.03^Ab^
NADH	0.18 ± 0.03^Aa^	0.40 ± 0.02^Ab^	0.35 ± 0.03^Ab^	0.38 ± 0.03^Ab^	0.42 ± 0.03^Ab^

The values are means ± SD of tree replicates and means marked by common letters are not statistically different at *p* ≤ 0.01 (uppercase) or *p* ≤ 0.05 (small letters) for values in the same row, according to Scheffè’s test performed between control and plants with different treatment.

**Table 2:** Mineral elements (µmol g^−1^ DW) in roots and leaves of *P. maritimum* irrigated with 50 and 100 mM of NaCl salt solution at 3 and 10 days.

	Ca	Na	K	Mg	Cu	Zn	Fe	Mn
Roots (µmol g^−1^DW)								
Control	50 ± 4^Bb^	240 ± 15^Dd^	400 ± 25^Aa^	165 ± 11^Aa^	0.95 ± 0.06^Cc^	2.50 ± 0.2^Cc^	25 ± 3^Cc^	6.5 ± 0.5^Bb^
50 mM 3d	45 ± 5^Bb^	380 ± 10^Ee^	350 ± 28^Ab^	150 ± 11^Aa^	0.90 ± 0.04^Cc^	2.80 ± 0.3^Cc^	22 ± 2^Cc^	7.1 ± 0.6^Bb^
50 mM 10 d	45 ± 6^Bb^	510 ± 18^Ff^	300 ± 32^Ab^	135 ± 11^Ab^	0.85 ± 0.05^Cc^	3.00 ± 0.2^Cc^	20 ± 2^Cc^	7.6 ± 0.7^Bb^
100 mM 3 d	40 ± 6^Bb^	524 ± 19^Gg^	280 ± 29^Ab^	130 ± 7^Ab^	0.88 ± 0.04^Cc^	3.10 ± 0.2^Cc^	21 ± 2^Cc^	7.5 ± 0.8^Bb^
100 mM 10d	29 ± 4^Cc^	890 ± 28^Hh^	170 ± 21^Cc^	110 ± 8^Ab^	0.65 ± 0.06^D^	3.50 ± 0.3^Cd^	17 ± 1^Cd^	8.5 ± 0.7^Bc^
Leaves (µmol g^−1^DW)								
Control	130 ± 6^Aa^	47 ± 5^Aa^	450 ± 28^Aa^	182 ± 20^Aa^	0.45 ± 0.07^Aa^	0.84 ± 0.05^Aa^	90 ± 5^Aa^	2.5 ± 0.2^Aa^
50 mM 3 d	120 ± 4^Aa^	63 ± 6^Bb^	430 ± 25^Aa^	175 ± 18^Aa^	0.40 ± 0.03^Aa^	0.90 ± 0.05^Aa^	85 ± 4^Aa^	2.7 ± 0.2^Aa^
50 mM 10d	100 ± 4^Ab^	80 ± 7^Bb^	420 ± 25^Aa^	155 ± 17^Aa^	0.35 ± 0.04^Ab^	0.96 ± 0.05^Aa^	80 ± 7^Aa^	3.0 ± 0.3^Aa^
100 mM 3d	110 ± 6^Aa^	75 ± 5^Bb^	425 ± 31^Aa^	160 ± 11^Aa^	0.40 ± 0.04^Aa^	0.98 ± 0.05^Aa^	80 ± 8^Aa^	3.2 ± 0.3^Aa^
100 mM 10d	90 ± 4^Ab^	110 ± 6^Cc^	400 ± 35^Aa^	130 ± 11^Ab^	0.30 ± 0.04^Ab^	1.30 ± 0.05^Bc^	70 ± 7^Ab^	3.5 ± 0.3^Ab^

The values (μmol g^−1^ DW) are means ± SD of three replicates. The values marked by common letters are not statistically different at *p* ≤ 0.01 (uppercase) or *p* ≤ 0.05 (small letters) for values in the same column, according to Scheffè’s test performed between control and plants with different treatment.

**Table 3:** Free amino acid concentrations (µmol g^−1^ FW) of roots and leaves of *P. maritimum* plants irrigated with 50 and 100 mM of NaCl salt solution for 3 and 10 days.

	Control	50 mM NaCl		100 mM NaCl	
Days	0	3	10	3	10
Roots (µmol g^−1^ FW)					
Glycine	0.30 ± 0.03^Aa^	0.35 ± 0.03^Aa^	0.45 ± 0.03^Ab^	0.35 ± 0.02^Aa^	0.50 ± 0.05^Ab^
Serine	0.20 ± 0.03^Aa^	0.20 ± 0.03^Aa^	0.35 ± 0.03^Ab^	0.25 ± 0.05^Aa^	0.40 ± 0.05^Ab^
Aspartate	1.00 ± 0.30^Aa^	1.50 ± 0.30^Aa^	1.50 ± 0.40^Aa^	1.40 ± 0.40^Aa^	1.70 ± 0.40^Aa^
Glutamate	1.80 ± 0.40^Aa^	3.50 ± 0.50^Ab^	3.20 ± 0.30^Ab^	3.70 ± 0.50^Ab^	3.20 ± 0.40^Ab^
Asparagine	0.25 ± 0.06^Aa^	0.50 ± 0.07^Ab^	0.50 ± 0.06^Ab^	0.50 ± 0.07^Ab^	0.70 ± 0.07^Ac^
Treonine	0.04 ± 0.005^Aa^	0.08 ± 0.01^Ab^	0.07 ± 0.01^Ab^	0.10 ± 0.02^Ab^	0.10 ± 0.02^Ab^
Arginine	0.40 ± 0.08^Aa^	0.60 ± 0.10^Ab^	0.60 ± 0.07^Ab^	0.50 ± 0.08^Aa^	0.60 ± 0.10^Ab^
Glutamine	1.00 ± 0.20^Aa^	2.00 ± 0.20^Ab^	2.20 ± 0.30^Ab^	2.50 ± 0.40^Ab^	2.40 ± 0.60^Ab^
Alanine	0.30 ± 0.05^Aa^	0.50 ± 0.06^Ab^	0.60 ± 0.10^Ab^	0.80 ± 0.09^Ab^	0.80 ± 0.10^Ab^
Tryptophan	0.05 ± 0.01^Aa^	0.12 ± 0.01^Ab^	0.10 ± 0.01^Ab^	0.15 ± 0.02^Ab^	0.13 ± 0.02^Ab^
Valine	0.04 ± 0.01^Aa^	0.07 ± 0.01^Ab^	0.08 ± 0.01^Ab^	0.10 ± 0.02^Ab^	0.10 ± 0.02^Ab^
Isoleucine	0.02 ± 0.004^Aa^	0.05 ± 0.01^Ab^	0.07 ± 0.01^Ab^	0.07 ± 0.01^Ab^	0.08 ± 0.01^Ab^
Leucine	0.04 ± 0.01^Aa^	0.08 ± 0.01^Ab^	0.08 ± 0.01^Ab^	0.10 ± 0.02^Ab^	0.09 ± 0.02^Ab^
Lysine	0.05 ± 0.01^Aa^	0.09 ± 0.03^Ab^	0.08 ± 0.02^Ab^	0.10 ± 0.02^Ab^	0.10 ± 0.02^Ab^
Citrulline	0.20 ± 0.04^Aa^	0.25 ± 0.03^Aa^	0.20 ± 0.03^Aa^	0.35 ± 0.02^Aa^	0.25 ± 0.02^Aa^
Proline	0.30 ± 0.05^Aa^	2.60 ± 0.40^Bb^	6.00 ± 1.00^Cc^	4.00 ± 0.70^Bb^	8.00 ± 0.60^Cc^
Σ AA	5.99 ± 0.70^Aa^	12.4 ± 1.50^Bb^	16.0 ± 0.80^Bc^	14.6 ± 1.20^Bc^	19.0 ± 1.50^Bd^
Glycine betaine	1.00 ± 0.17^Aa^	1.00 ± 0.02^Aa^	4.00 ± 0.7^Bb^	1.30 ± 0.30^Aa^	6.00 ± 0.70^Bc^
Leaves (µmol g^−1^ FW)					
Glycine	0.60 ± 0.06^Aa^	0.90 ± 0.08^Bb^	1.80 ± 0.40^Cc^	1.60 ± 0.30^Cc^	2.50 ± 0.30^Dd^
Serine	0.70 ± 0.10^Aa^	0.80 ± 0.1.0^Aa^	1.60 ± 0.30^Bb^	1.20 ± 0.20^Bb^	2.00 ± 0.30^Cc^
Aspartate	0.80 ± 0.20^Aa^	1.00 ± 0.04^Aa^	1.00 ± 0.08^Aa^	1.30 ± 0.05^Aa^	1.20 ± 0.03^Aa^
Glutamate	2.40 ± 0.30^Aa^	4.00 ± 0.70^Bb^	4.00 ± 1.00^Bb^	4.50 ± 0.20^Bb^	4.00 ± 0.90^Bb^
Asparagine	0.45 ± 0.03^Aa^	0.60 ± 0.10^Ab^	0.65 ± 0.02^Ab^	0.65 ± 0.02^Ab^	0.65 ± 0.03^Ab^
Treonine	0.05 ± 0.01^Aa^	0.07 ± 0.01^Aa^	0.08 ± 0.02^Aa^	0.09 ± 0.02^Aa^	0.10 ± 0.02^Aa^
Arginine	0.60 ± 0.10^Aa^	0.90 ± 0.10^Ab^	1.00 ± 0.20^Ab^	0.80 ± 0.10^Ab^	1.10 ± 0.10^Ab^
Glutamine	1.50 ± 0.25^Aa^	3.00 ± 0.70^Bb^	2.80 ± 0.20^Bb^	3.20 ± 0.33^Bb^	3.00 ± 0.20^Bb^
Alanine	0.50 ± 0.08^Aa^	0.70 ± 0.08^Aa^	0.90 ± 0.10^Aa^	0.90 ± 0.20^Aa^	1.20 ± 0.30^Aa^
Tryptophan	0.05 ± 0.01^Aa^	0.07 ± 0.02^Aa^	0.09 ± 0.01^Aa^	0.09 ± 0.01^Aa^	0.10 ± 0.02^Aa^
Valine	0.04 ± 0.01^Aa^	0.07 ± 0.01^Ab^	0.08 ± 0.01^Ab^	0.07 ± 0.01^Aa^	0.07 ± 0.01^Ab^
Isoleucine	0.04 ± 0.01^Aa^	0.07 ± 0.02^Ab^	0.08 ± 0.08^Ab^	0.07 ± 0.07^Ab^	0.09 ± 0.01^Ab^
Leucine	0.05 ± 0.01^Aa^	0.08 ± 0.02^Aa^	0.07 ± 0.01^Aa^	0.09 ± 0.01^Aa^	0.06 ± 0.01^Ab^
Lysine	0.06 ± 0.01^Aa^	0.10 ± 0.02^Ab^	0.12 ± 0.01^Ab^	0.12 ± 0.01^Ab^	0.14 ± 0.01^Ab^
Citrulline	0.20 ± 0.05^Aa^	0.35 ± 0.03^Ab^	0.45 ± 0.05^Ab^	0.30 ± 0.05^Ab^	0.40 ± 0.06^Ab^
Proline	0.70 ± 0.10^Aa^	7.00 ± 0.8^Bb^	15.0 ± 0.80^Cc^	11.0 ± 1.50^Bd^	20.0 ± 2.00^De^
Σ AA	8.70 ± 0.70^Aa^	19.4 ± 1.40^Bb^	29.7 ± 1.90^Cc^	25.7 ± 1.60^Cd^	37.5 ± 1.70^De^
Glycine betaine	1.50 ± 0.20^Aa^	1.70 ± 0.10^Aa^	5.00 ± 0.80^Ab^	1.60 ± 0.05^Aa^	6.50 ± 1.00^Ab^

The values are means ± SD of three replicates. The values marked by common letters are not statistically different at *p* ≤ 0.01. (uppercase) or *p* ≤ 0.05 (small letters) for values in the same row, according to Scheffè’s test performed between control and plants with different treatment.

## Data Availability

Not applicable.

## Supplementary Materials

The following are available online at https://www.mdpi.com/2223-7747/10/2/345/s1, Figure S1: GSH/GSSG ratio in roots (light green) and leaves (brilliant green) tissue of *P. maritimum* plants irrigated with 50 mM (wide striped) and 100 mM (narrow striped) of NaCl salt solution for 3 and 10 days, Figure S2: Spearman positive correlation of metabolites and OAS-TL activity in *P. maritimum* under salt treatment (50 and 100 mM) for 3 and 10 days.
