# Supplementary material for: Simultaneous Biochemical and Physiological Responses of the Roots and Leaves of Pancratium maritimum (Amaryllidaceae) to Mild Salt Stress
Source: Plants (Basel). 2021 Feb 11;10(2):345. doi: 10.3390/plants10020345 (PMC7918514; doi:10.3390/plants10020345)
Supplement: Supplementary file 1 [file plants-10-00345-s001.pdf]

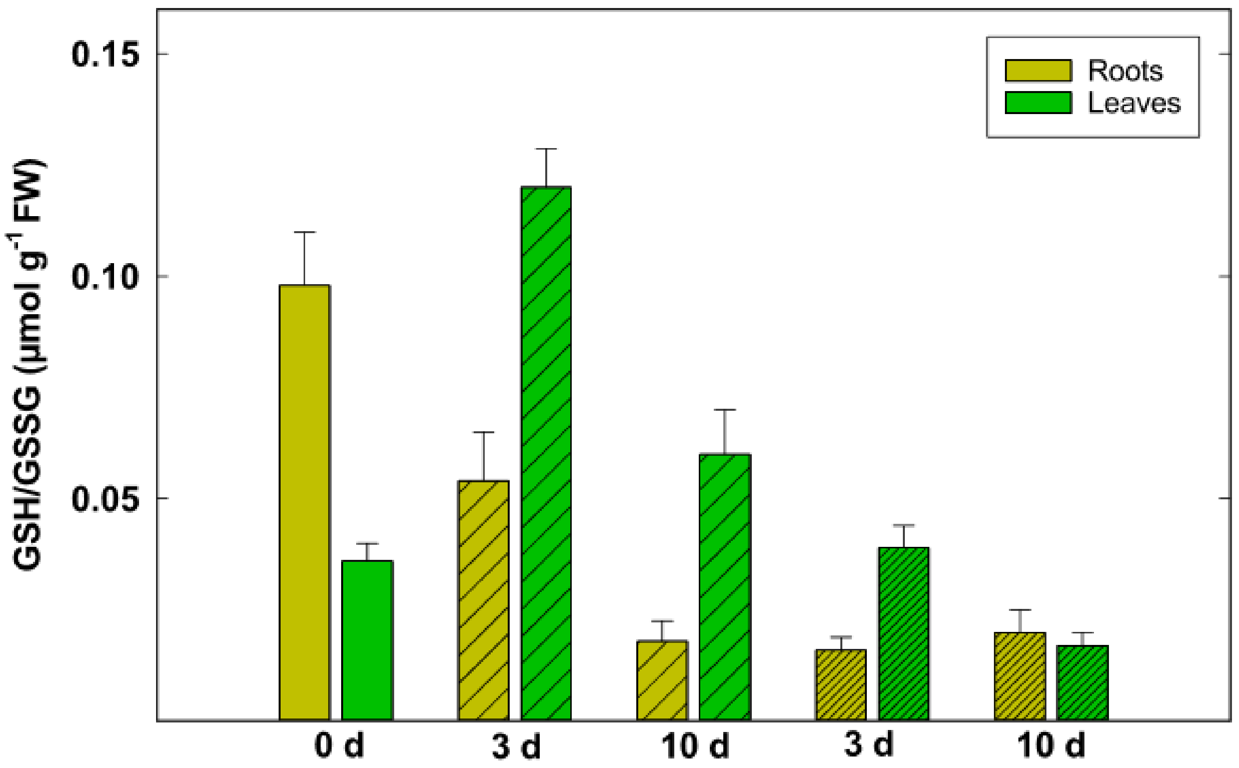

Figure S1. GSH/GSSG ratio in roots (light green) and leaves (brilliant green) tissue of *P. maritimum* plants irrigated with 50 mM (wide striped) and 100 mM (narrow striped) of NaCl salt solution for 3 and 10 days (d). The values are means ± SD of three replicates.

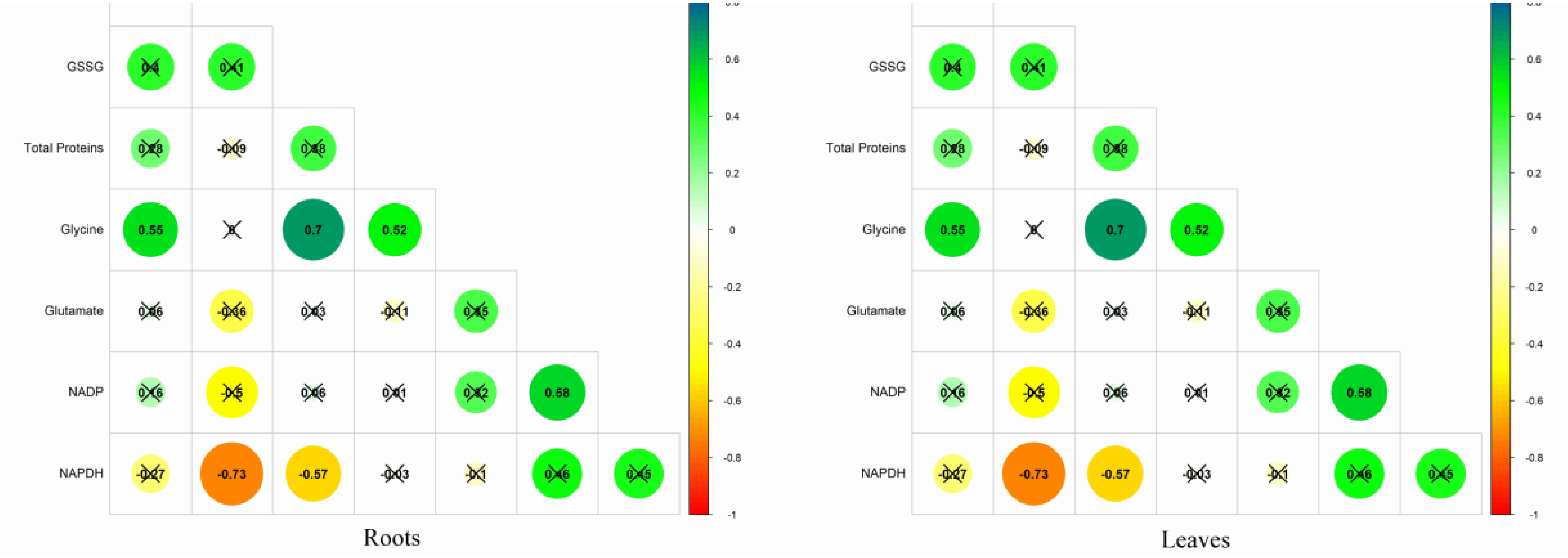

Figure S2. Spearman positive correlation of metabolites and OAS-TL activity in *P. maritimum* under salt treatment (50 and 100 mM) for 3 and 10 days.

Table S1. Ratio of salts concentration (µmol g^{-1} DW) in the leaves and roots (L:R) in *P. maritimum* plants irrigated with 50 mM and 100 mM of NaCl salt solution for 3 and 10 days (d). The values are means ± SD of three replicates.

	**Ca**	**Na**	**K**	**Mg**	**Cu**	**Zn**	**Fe**	**Mn**
**Control**	2.60 ± 0.10	0.19 ± 0.009	1.12 ± 0.02	1.11 ± 0.20	0.48 ± 0.10	0.34 ± 0.05	3.64 ± 0.36	0.38 ± 0.03
**50 mM 3d**	2.69 ± 0.23	0.18 ± 0.02	1.24 ± 0.18	1.13 ± 0.18	0.45 ± 0.17	0.32 ± 0.05	3.90 ± 0.66	0.38 ± 0.03
**50 mM 10 d**	2.22 ± 0.17	0.16 ± 0.018	1.35 ± 0.18	1.14 ± 0.07	0.42 ± 0.06	0.33 ± 0.03	4.08 ± 0.61	0.39 ± 0.03
**100 mM 3 d**	2.46 ± 0.59	0.14 ± 0.012	1.43 ± 0.37	1.22 ± 0.02	0.45 ± 0.03	0.31 ± 0.04	3.81 ± 0.46	0.43 ± 0.08
**100 mM 10d**	3.10 ± 0.28	0.12 ± 0.009	2.37 ± 0.12	1.19 ± 0.11	0.46 ± 0.07	0.36 ± 0.06	4.28 ± 0.98	0.41 ± 0.06
